# Cigarette Smoke Extract Inhibits Platelet Aggregation by Suppressing Cyclooxygenase Activity

**DOI:** 10.1055/s-0037-1607979

**Published:** 2017-10-30

**Authors:** Hitoshi Kashiwagi, Koh-ichi Yuhki, Yoshitaka Imamichi, Fumiaki Kojima, Shima Kumei, Tsunehito Higashi, Takahiro Horinouchi, Soichi Miwa, Shuh Narumiya, Fumitaka Ushikubi

**Affiliations:** 1 Department of Pharmacology, Asahikawa Medical University, Asahikawa, Japan; 2 Core Research for Evolutional Science and Technology, Japan Science and Technology Agency, Tokyo, Japan; 3 Department of Pharmacology, Kitasato University, Sagamihara, Japan; 4 Department of Cellular Pharmacology, Hokkaido University Graduate School of Medicine, Sapporo, Japan; 5 Medical Innovation Center, Kyoto University Graduate School of Medicine, Kyoto, Japan

**Keywords:** cigarette smoke extract, platelets, thromboxane A _2_, cyclooxygenase

## Abstract

The results of studies that were performed to determine whether cigarette smoking affects platelet function have been controversial, and the effects of nicotine- and tar-free cigarette smoke extract (CSE) on platelet function remain to be determined. The aim of this study was to determine the effect of CSE on platelet aggregation and to clarify the mechanism by which CSE affects platelet function. CSE inhibited murine platelet aggregation induced by 9,11-dideoxy-9α,11α-methanoepoxy-prosta-5Z,13E-dien-1-oic acid (U-46619), a thromboxane (TX) A _2_ receptor agonist, and that induced by collagen with respective IC _50_ values of 1.05 ± 0.14% and 1.34 ± 0.19%. A similar inhibitory action of CSE was also observed in human platelets. CSE inhibited arachidonic acid–induced TXA _2_ production in murine platelets with an IC _50_ value of 7.32 ± 2.00%. Accordingly, the inhibitory effect of CSE on collagen-induced aggregation was significantly blunted in platelets lacking the TXA _2_ receptor compared with the inhibitory effect in control platelets. In contrast, the antiplatelet effects of CSE in platelets lacking each inhibitory prostanoid receptor, prostaglandin (PG) I _2_ receptor and PGE _2_ receptor subtypes EP _2_ and EP _4_ , were not significantly different from the effects in respective control platelets. Among the enzymes responsible for TXA _2_ production in platelets, the activity of cyclooxygenase (COX)-1 was inhibited by CSE with an IC _50_ value of 1.07 ± 0.15% in an uncompetitive manner. In contrast, the activity of TX synthase was enhanced by CSE. The results indicate that CSE inhibits COX-1 activity and thereby decreases TXA _2_ production in platelets, leading to inhibition of platelet aggregation.

## Introduction

Cigarette smoking has been recognized as a risk factor for various diseases including cancers, cardiovascular diseases, and respiratory diseases. 1 2 In the development of cardiovascular diseases, platelets are known to play an important role by both aggregating themselves and releasing various bioactive substances such as growth factors, lysophospholipids, and chemokines. 3 4 5 6 Accordingly, a large number of studies have been performed to investigate the effect of cigarette smoke on platelets. Cigarette smoking potentiated platelet aggregation induced by various stimulants, 7 8 and platelet activity was increased by both the mainstream and sidestream of cigarette smoke. 9 Furthermore, activated platelets from smokers showed spontaneous aggregation 10 and contributed to the augmented strength of blood clots. 11 Among smokers, platelet aggregation was more enhanced in long-term smokers than in short-term smokers. 12 However, some studies showed an inhibitory effect or no effect of cigarette smoking on platelet function. It was shown that the degree of platelet aggregation in response to collagen was lower in habitual smokers than in nonsmokers. 13 In addition, it was shown that platelet aggregation induced by various stimulants and platelet hemostatic capacity were decreased or unchanged in smokers compared with those in nonsmokers. 14 Thus, the effect of cigarette smoking on platelet function remains to be determined.

Nicotine is the representative bioactive agent contained in cigarette smoke, and the effect of nicotine on platelets has been investigated in some studies. Nicotine potentiated thrombin- or adenosine diphosphate (ADP)-induced platelet aggregation. 7 15 In addition to nicotine, at least 4,800 chemical constituents have been identified in cigarette smoke, 16 17 and some of them have been shown to affect platelet function, 18 19 20 possibly being one of the reasons why the effect of cigarette smoking on platelets is complicated. Cigarette smoke is a complex aerosol consisting of a particulate phase and a gas phase. Among the constituents of the particulate phase, tar is defined by the Federal Trade Commission as the total particulate matter of cigarette smoke other than nicotine and water. Various bioactive substances such as carbon monoxide, carbon dioxide, nitrogen oxides, formaldehyde, acetone, and acrolein have also been found in the gas phase. 16 21

In this study, we attempted to determine the effect of nicotine- and tar-free cigarette smoke extract (CSE) on platelets and to clarify the mechanism by which CSE affects platelet function. We first examined the effect of CSE on platelet aggregation. Then we investigated whether CSE can change thromboxane (TX) A _2_ production and intracellular concentration of cyclic adenosine monophosphate (cAMP) in platelets. Finally, we examined whether CSE affects activities of cyclooxygenase (COX) and TX synthase, enzymes responsible for TXA _2_ production.

## Materials and Methods

### Materials

U-46619 and arachidonic acid were purchased from Cayman Chemical (Ann Arbor, Michigan, United States). Collagen and ADP were purchased from Nycomed Pharma (Munich, Germany) and Sigma-Aldrich (St. Louis, Missouri, United States), respectively. Cigarettes were purchased from Japan Tobacco Inc. (Tokyo, Japan), and the cigarette brand used in this study was Hi-Lite, in which one cigarette contains 1.4 mg nicotine and 17 mg tar. Cambridge glass fiber filters were purchased from Heinrich Borgwaldt GmbH (Hamburg, Germany). 3-Isobutyl-1-methylxanthine (IBMX) was purchased from Sigma-Aldrich.

### Mice

Generation and maintenance of mice lacking the prostaglandin (PG) E _2_ receptor subtype EP _2_ (EP _2_^–/–^ mice) or EP _4_ (EP _4_^–/–^ mice), PGI _2_ receptor IP (IP ^–/–^ mice), or TXA _2_ receptor TP (TP ^–/–^ mice) have been reported. 22 23 24 25 These mice and wild-type (WT) control mice, but with the exception of EP _4_^–/–^ mice, have a genetic background of C57BL/6 mice. EP _4_^–/–^ mice have a mixed genetic background of C57BL/6 and 129/Ola mice. 23 For the experiments using EP _4_^–/–^ mice, F2-WT mice having a similar genetic background were used as controls. All the experiments, which were approved by the Asahikawa Medical University Committee on Animal Research, were performed on 11- to 23-week-old male mice.

### Cigarette Smoke Extract Preparation

Nicotine- and tar-free CSE was prepared according to a previously reported method 26 with a slight modification. Briefly, the mainstream smoke of each cigarette was aspirated at a flow rate of 1.05 L/minute and passed through a Cambridge glass fiber filter to remove the particulate phase constituents. The filtered smoke was bubbled into phosphate-buffered saline (PBS) at 25°C, and this procedure was repeated. The gas phase constituents of smoke from 10 cigarettes were dissolved in 10 mL of PBS, and this solution was defined as 100% CSE.

### Platelet Preparation

Platelet-rich plasma (PRP) and washed platelets of mice were prepared as reported previously 27 with a slight modification. Briefly, blood was drawn by cardiac puncture from isoflurane-anesthetized mice and was diluted immediately with an equal volume of an experimental buffer (20 mM HEPES, 140 mM NaCl, 5 mM MgCl _2_ , and 5 mM KCl, pH 7.4). Diluted blood was then centrifuged at 90 *g* for 5 minutes, and PRP was obtained by collecting the upper phase. Platelet-poor plasma (PPP) was prepared by further centrifuging the remaining lower phase at 1,500 *g* for 10 minutes. In platelet aggregation studies, the number of platelets in PRP was adjusted to 3 × 10 ^5^ platelets/μL with PPP, and the final concentration of trisodium citrate was adjusted to 0.38%. To prepare washed platelets, a one-tenth volume of 77 mM EDTA (pH 7.4) was added to PRP and the mixture was centrifuged at 900 *g* for 15 minutes. The platelet pellet was washed once with a washing buffer (135 mM NaCl, 5 mM KCl, 8 mM Na _2_ HPO _4_ , 2 mM NaH _2_ PO _4_ , and 10 mM EDTA, pH 7.2) and centrifuged at 900 *g* for 15 minutes. Finally, the platelet pellet was suspended in modified Tyrode's buffer (10 mM HEPES, 137 mM NaCl, 2.7 mM KCl, 12 mM NaHCO _3_ , 0.42 mM NaH _2_ PO _4_ , and 1 mM MgCl _2_ , pH 7.4) and the number of platelets was adjusted to 10 ^6^ platelets/μL. For human platelet preparation, informed consent was obtained from healthy volunteers who had not taken any medications and cigarettes for at least the past 14 days. Blood was drawn by venepuncture and was then centrifuged at 140 *g* for 10 minutes. PRP was obtained by collecting the upper phase and PPP was prepared by further centrifuging the remaining lower phase at 1,500 *g* for 15 minutes. The number of platelets in PRP was adjusted to 3 × 10 ^5^ platelets/μL with PPP, and the final concentration of trisodium citrate was adjusted to 0.38%. The experiments using human platelets were approved by the Asahikawa Medical University Research Ethics Committee.

### Platelet Aggregation Study

Platelet aggregation was examined by a previously reported method using an aggregometer (PAT-4A, Nihon Kohden, Tokyo, Japan) 27 with a slight modification. Briefly, PRP (200 μL) being stirred at 37°C in a cuvette was preincubated for 5 minutes and then CSE was added to the PRP 1 minute before the addition of U-46619 (a TP agonist), collagen, or ADP. Unstimulated PRP and PPP were set to show 0 and 100% of light transmission, respectively, and a peak of light transmission represents platelet aggregation. U-46619 was added at a concentration to induce murine platelet aggregation of 40 to 55% (2.5–3.5 μM). Collagen and ADP were added at concentrations to induce murine platelet aggregation of 40 to 55% (1.0–1.5 μg/mL) and 45 to 55% (3.5–4.5 μM), respectively. When using PRP prepared from TP ^–/–^ mice, the concentration of collagen was increased (2.5–3.5 μg/mL) to induce a degree of aggregation similar to that in PRP prepared from WT mice. For human platelets, U-46619 and collagen were added at concentrations to induce aggregation of 45 to 50% (0.5–0.8 μM) and 45 to 55% (0.5–1.0 μg/mL), respectively.

### Measurement of TXB _2_ Content

Washed platelets (100 μL) were preincubated in the absence of calcium and fibrinogen for 5 minutes at 37°C, and then CSE was added 5 minutes before the addition of 1 μM arachidonic acid or 10 nM PGH _2_ . After further incubation for 5 minutes at 37°C, the reaction was terminated by the addition of ice-cold 1 N HCl (25 μL), and the platelet suspension was centrifuged at 20,400 *g* for 10 minutes at 4°C. The supernatant was neutralized with a one-fifth volume of 1 M Tris (pH 10.4), and the content of TXB _2_ was measured by using a TXB _2_ enzyme immunoassay Kit (Cayman Chemical).

### Measurement of cAMP Production

Washed platelets (100 μL) were preincubated in the presence of 1 mM IBMX, an inhibitor of phosphodiesterase, for 10 minutes at 37°C, and then CSE was added. After further incubation for 10 minutes at 37°C, the reaction was terminated by the addition of 30% trichloroacetic acid (25 μL), and the platelets were disrupted by sonication. The solution was centrifuged at 20,400 *g* for 10 minutes at 4°C. The content of cAMP in the supernatant was determined by using a radioimmunoassay kit (Yamasa Shoyu, Chiba, Japan) after trichloroacetic acid had been extracted three times with water-saturated diethyl ether.

### Measurement of COX Activity

COX-1 and COX-2 activities were measured using a COX Fluorescent Inhibitor Screening Assay Kit (Cayman Chemical). Briefly, CSE was added to a solution containing ovine COX-1 or human COX-2, and the mixture was incubated for 5 minutes at room temperature. To initiate the reaction, arachidonic acid was added to the mixture, and then the sample was further incubated at room temperature. After 2-minute incubation, the fluorescence was measured with an excitation wavelength of 535 nm and an emission wavelength of 590 nm using a microplate reader (Synergy H1; BioTek, Winooski, Vermont, United States). To determine the manner of the inhibitory action of CSE on COX-1, we examined the effect of CSE (3%) on the Michaelis constant (K _m_ ) and the maximum velocity (V _max_ ) of COX-1 within the Michaelis–Menten kinetics.

### Data Analysis

**Fig. 1:**
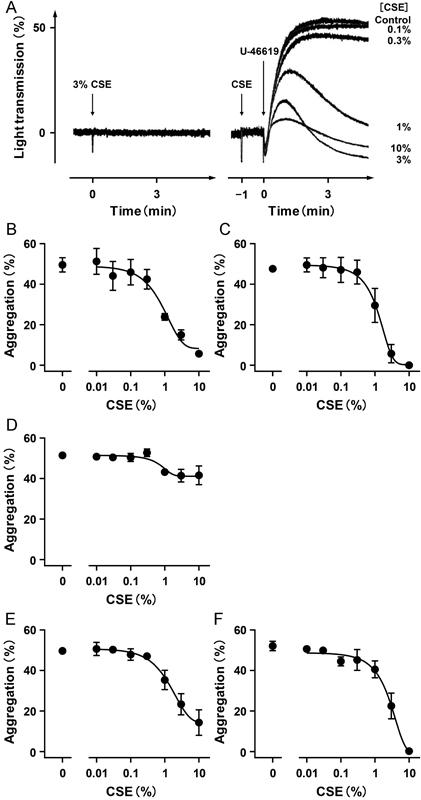
Effects of cigarette smoke extract (CSE) on platelet aggregation. ( **A** ) CSE was added at the indicated concentration to PRP alone (left) or 1 minute before the addition of 3 μM U-46619 (right). A decrease and an increase in light transmission indicate platelet shape change and platelet aggregation, respectively. Similar results were obtained in five independent PRP preparations, and representative results are shown. Control, no addition of CSE. U-46619 ( **B** ), collagen ( **C** ), and ADP ( **D** ) were added at concentrations to induce murine platelet aggregation of 40 to 55% (2.5–3.5 μM), 40 to 55% (1.0–1.5 μg/mL), and 45 to 55% (3.5–4.5 μM), respectively. CSE was added at the indicated concentration to PRP 1 minute before the addition of each stimulant. Each value is the mean ± SEM. ( **B** ) *n* = 5; ( **C** ) *n* = 6; ( **D** ) *n* = 5. U-46619 ( **E** ) and collagen ( **F** ) were added at concentrations to induce human platelet aggregation of 45 to 50% (0.5–0.8 μM) and 45 to 55% (0.5–1.0 μg/mL), respectively. CSE was added at the indicated concentration to PRP 1 minute before the addition of each stimulant. Each value is the mean ± SEM, *n* = 4.

All data except those shown in Fig. 1A are expressed as means ± standard error of the mean (SEM). Statistical comparisons of data were made by repeated two-way analysis of variance. A *p* -value of < 0.05 was considered statistically significant.

## Results

### CSE Inhibits U-46619- or Collagen-Induced Platelet Aggregation

To determine whether cigarette smoke components other than nicotine and tar affect platelet aggregation, we examined the effect of nicotine- and tar-free CSE on aggregation in platelets prepared from WT mice (WT platelets). At a concentration of 3%, CSE alone did not induce platelet aggregation or platelet shape change ( Fig. 1A , left). Next, we examined whether CSE affects U-46619-induced platelet aggregation. CSE inhibited U-46619-induced platelet aggregation in a concentration-dependent manner with an IC _50_ value of 1.05 ± 0.14% ( Fig. 1A [right], B ). CSE also inhibited collagen-induced platelet aggregation in a concentration-dependent manner with an IC _50_ value of 1.34 ± 0.19% ( Fig. 1C ). On the other hand, the inhibitory effect of CSE on ADP-induced platelet aggregation was weak, and its inhibition was only 19.1 ± 6.3% of control aggregation at a maximum concentration ( Fig. 1D ). CSE also inhibited U-46619- and collagen-induced aggregation of human platelets with potencies similar to those in murine platelets; the IC _50_ values were 2.80 ± 0.37% and 2.65 ± 0.26%, respectively ( Fig. 1E , F ).

### CSE Inhibits TXA _2_ Production in Platelets, and Its Inhibitory Action on Collagen-Induced Aggregation Is Attenuated in Platelets Lacking the TXA _2_ Receptor

**Fig. 2:**
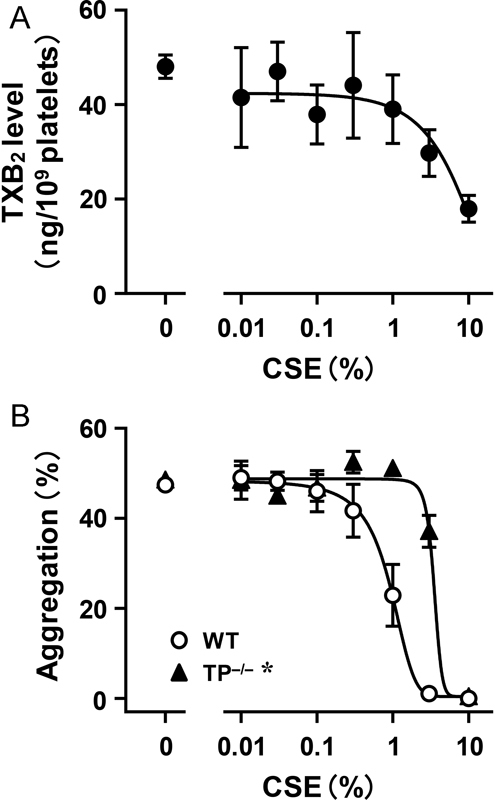
Contribution of reduction in TXA _2_ production to the inhibitory effect of cigarette smoke extract (CSE) on platelet aggregation. ( **A** ) To estimate the degree of TXA _2_ production in platelets, we measured the content of TXB _2_ , a stable TXA _2_ metabolite. Arachidonic acid (1 μM) was added to washed platelets to induce TXA _2_ production and incubated for 5 minutes. CSE was added at the indicated concentration to washed platelets 5 minutes before the addition of arachidonic acid. Each value is the mean ± SEM, *n* = 9. ( **B** ) Effects of CSE on collagen-induced aggregation of platelets prepared from WT and TP ^–/–^ mice. Collagen was added at a concentration to induce platelet aggregation of 40 to 55% (WT, 1.0–1.5 μg/mL; TP ^–/–^ , 2.5–3.5 μg/mL). CSE was added at the indicated concentration to PRP 1 minute before the addition of collagen. Each value is the mean ± SEM. WT, *n* = 5; TP ^–/–^ , *n* = 6. * *p* < 0.05 versus WT.

TXA _2_ is known as a potent stimulator of platelets, and it is produced when platelets are activated. Therefore, it works as a central regulator of platelet activation in a positive feedback manner. To determine whether CSE affects TXA _2_ production in platelets, we examined the effect of CSE on arachidonic acid-induced TXA _2_ production. For assessment of the degree of TXA _2_ production, we measured the content of TXB _2_ , a stable TXA _2_ metabolite. To exclude TXA _2_ production associated with platelet aggregation, we performed the experiment in a buffer without calcium and fibrinogen, which enable aggregation. Under this condition, CSE inhibited TXA _2_ production in a concentration-dependent manner with an IC _50_ value of 7.32 ± 2.00% ( Fig. 2A ). To confirm that the reduced production of TXA _2_ contributes to the inhibitory action of CSE, we examined the effect of CSE on collagen-induced aggregation in platelets lacking the TXA _2_ receptor TP (TP ^–/–^ platelets). Since TP-mediated signaling plays a role in collagen-induced platelet aggregation, the concentration of collagen was increased to induce a degree of aggregation in TP ^–/–^ platelets similar to that in WT platelets. In TP ^–/–^ platelets, the inhibitory effect of CSE on collagen-induced aggregation was attenuated significantly compared with that in WT platelets; the IC _50_ values were 3.51 ± 0.13% and 0.92 ± 0.15%, respectively ( Fig. 2B ). These results indicate that TXA _2_ -mediated signaling contributes to the inhibitory action of CSE on platelet aggregation.

### Prostanoid Receptors Other Than TP Do Not Participate in the Inhibitory Action of CSE on Platelet Aggregation, and CSE Does Not Affect Intraplatelet cAMP Concentration

**Fig. 3:**
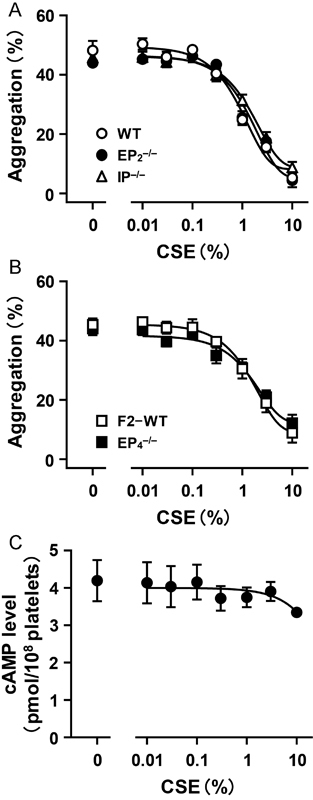
Nonparticipation of inhibitory prostanoid receptors in the inhibitory effect of cigarette smoke extract (CSE) on platelet aggregation. ( **A, B** ) Effects of CSE on U-46619-induced aggregation in platelets prepared from WT, EP _2_^–/–^ , and IP ^–/–^ mice ( **A** ) and from F2-WT and EP _4_^–/–^ mice ( **B** ). U-46619 was added at a concentration to induce platelet aggregation of 40 to 55% (2.5–3.5 μM). CSE was added at the indicated concentration to PRP 1 minute before the addition of U-46619. Each value is the mean ± SEM. WT, *n* = 6; EP _2_^–/–^ , *n* = 7; IP ^–/–^ , *n* = 6; F2-WT, *n* = 5; EP _4_^–/–^ , *n* = 8. ( **C** ) Effects of CSE on cAMP production in platelets. IBMX (1 mM) was added to washed platelets 10 minutes before the addition of CSE, which was added at the indicated concentration and incubated for 10 minutes. Each value is the mean ± SEM, *n* = 4.

It is well known that PGI _2_ plays a central role opposing TXA _2_ in the regulation of platelet function; stimulation of the PGI _2_ receptor IP leads to inhibition of platelet aggregation. 28 In addition, we found in a previous study that selective agonists for PGE _2_ receptor subtypes EP _2_ and EP _4_ potently inhibit platelet aggregation. 29 To determine whether these prostanoid receptors contribute to the inhibitory action of CSE on platelet aggregation, we examined the effect of CSE on U-46619-induced aggregation of platelets lacking EP _2_ (EP _2_^–/–^ platelets), EP _4_ (EP _4_^–/–^ platelets), or IP (IP ^–/–^ platelets). U-46619 induced similar degrees of aggregation in these platelets as well as WT platelets and platelets prepared from F2-WT mice (F2-WT platelets). In both EP _2_^–/–^ and IP ^–/–^ platelets, the inhibitory effects of CSE on U-46619-induced aggregation were not significantly different from the inhibitory effect in WT platelets. The IC _50_ values were 1.35 ± 0.14%, 1.46 ± 0.16%, and 1.14 ± 0.10%, respectively ( Fig. 3A ). In EP _4_^–/–^ platelets, the inhibitory effect of CSE was also not significantly different from that in F2-WT platelets. The IC _50_ values were 1.31 ± 0.15% and 1.42 ± 0.18%, respectively ( Fig. 3B ). These results indicate that these prostanoid receptors do not participate in the inhibitory action of CSE on platelet aggregation.

To confirm that CSE does not affect the signaling of the inhibitory prostanoid receptors, IP, EP _2_ , and EP _4_ , we examined whether CSE affects the intraplatelet concentration of cAMP, a second messenger of the inhibitory prostanoid receptors. CSE had no significant effect on the cAMP concentration in washed platelets prepared from WT mice ( Fig. 3C ), indicating that the signaling of the inhibitory prostanoid receptors does not participate in the inhibitory action of CSE.

### CSE Inhibits Both COX-1 and COX-2 Activities

**Fig. 4:**
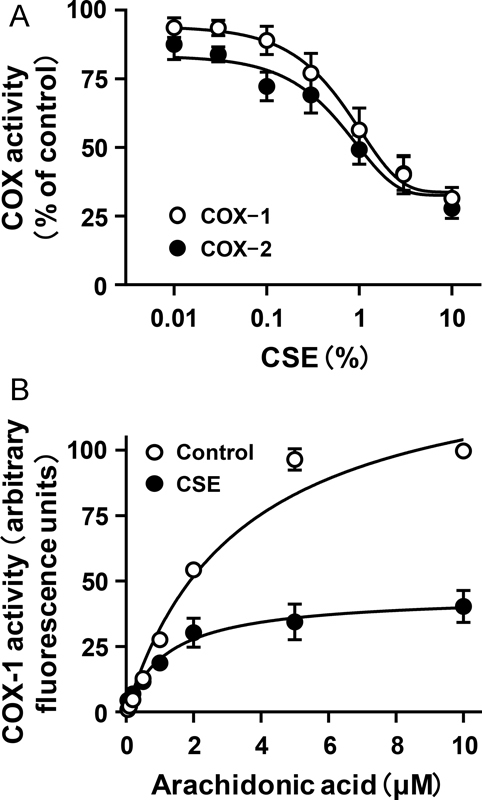
Inhibitory effects of cigarette smoke extract (CSE) on COX-1 and COX-2 activities. ( **A** ) Arachidonic acid was added to a solution containing ovine COX-1 or human COX-2 and incubated for 2 minutes. CSE was added at the indicated concentration to COX-1 or COX-2 5 minutes before the addition of arachidonic acid. Each value is the mean ± SEM, *n* = 5. ( **B** ) Arachidonic acid was added at the indicated concentration to a solution containing ovine COX-1 and incubated for 2 minutes. CSE (3%) was added to COX-1 5 minutes before the addition of arachidonic acid. Each value is the mean ± SEM, *n* = 4.

Since TXA _2_ synthesis was suppressed by CSE ( Fig. 2A ), we next examined whether CSE affects the activity of COX, a rate-limiting enzyme in the biosynthesis of prostanoids. COX has two isoforms, constitutive (COX-1) and inducible (COX-2) isoforms. CSE inhibited both COX-1 and COX-2 activities in a concentration-dependent manner, with respective IC _50_ values of 1.07 ± 0.15% and 0.80 ± 0.12% ( Fig. 4A ). To determine further the pattern of the inhibitory action of CSE, we analyzed the kinetics of the inhibitory action on COX-1, which is responsible for TXA _2_ synthesis in platelets. It has been shown that COX-2 was expressed in only a few platelets prepared from healthy donors. 30 CSE decreased K _m_ of COX-1 from 3.37 ± 0.40 to 1.21 ± 0.41 μM and also decreased V _max_ from 139.1 ± 6.8 to 44.6 ± 4.6 arbitrary fluorescence units ( Fig. 4B ), indicating that the pattern of COX-1 inhibition by CSE is uncompetitive.

### CSE Enhances TX Synthase Activity in Platelets

**Fig. 5:**
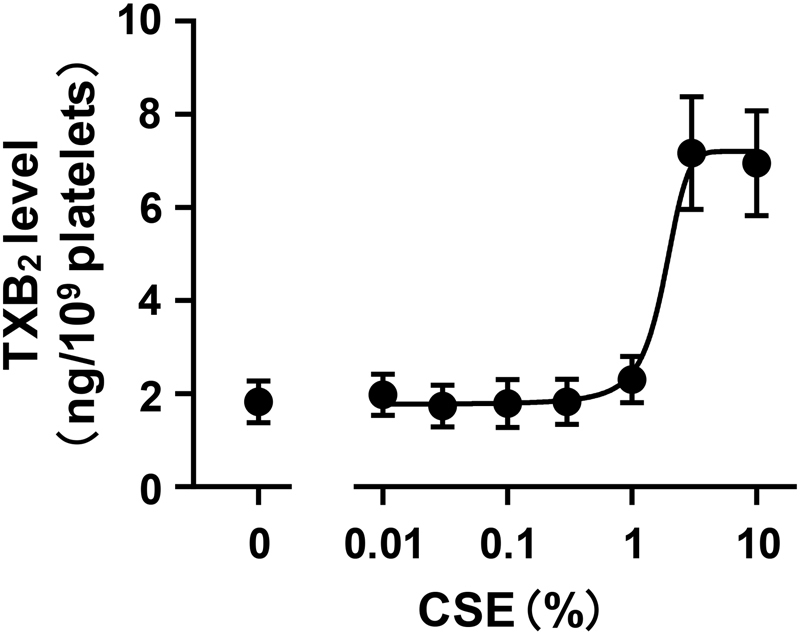
Potentiating effects of cigarette smoke extract (CSE) on TX synthase activity in platelets. To estimate the TX synthase activity in platelets, we added PGH _2_ to platelets and measured the content of TXB _2_ , a stable TXA _2_ metabolite. PGH _2_ (10 nM) was added to washed platelets to induce TXA _2_ production and incubated for 5 minutes. CSE was added at the indicated concentration to washed platelets 5 minutes before the addition of PGH _2_ . Each value is the mean ± SEM, *n* = 7.

TX synthase works downstream of COX, converting PGH _2_ to TXA _2_ . We finally examined whether CSE affects the activity of TX synthase. To assess TX synthase activity, we added PGH _2_ to the platelet suspension and examined the increase in the content of the stable TXA _2_ metabolite TXB _2_ in the medium. In contrast to the effect on COXs, CSE enhanced TXA _2_ synthase activity in a concentration-dependent manner with an EC _50_ value of 1.80 ± 0.57% ( Fig. 5 ).

## Discussion

Previous studies have shown that cigarette smoking is a major risk factor for cardiovascular diseases. 31 Platelet activation is one of the factors involved in the development of cardiovascular diseases. Activation levels of platelets are higher in smokers than in nonsmokers, and increased platelet activity leads to endothelial dysfunction, facilitating the development of atherosclerosis. 32 Although the precise mechanisms leading to platelet activation in smokers remain to be clarified, metabolic alterations found in smokers, such as increased plasma levels of fibrinogen 33 and catecholamine, 34 have been suggested to participate in platelet activation. Accordingly, thrombin- or ADP-induced aggregation in platelets prepared from nonsmokers was potentiated by the addition of plasma prepared from smokers. 35 Otherwise, the vascular endothelium is damaged due to the cytotoxic effect of cigarette smoke constituents, and endothelial PGI _2_ production is reduced, 36 which will likely lead to the activation of platelets. The direct actions of cigarette smoke on platelets have also been investigated in many studies. However, the results are contradictory, 9 15 and the direct effects of cigarette smoke remain to be elucidated.

We first examined the effect of nicotine- and tar-free CSE on platelet aggregation. CSE potently inhibited murine platelet aggregation induced by U-46619 or collagen ( Fig. 1A – C ). This result is consistent with the results of a previous study showing an inhibitory effect of filtered extract of cigarette smoke on platelet adhesion to collagen and fibrinogen. 15 Notably, the inhibitory effect of CSE on U-46619- or collagen-induced aggregation was also observed in human platelets ( Fig. 1E , F ). In contrast to the case of U-46619- or collagen-induced platelet aggregation, the inhibitory effect of CSE on ADP-induced aggregation was very weak ( Fig. 1D ), indicating that the inhibitory action of CSE on U-46619- or collagen-induced platelet aggregation does not result from its cytotoxicity. Our results for ADP-induced platelet aggregation differ from the results of a previous study showing that filtered gas phase extract inhibited ADP-induced aggregation in human and rabbit platelets. 37 Although the reason of this difference in the effects of CSE on ADP-induced platelet aggregation remains to be clarified, the reason may be the species difference.

We next intended to clarify the mechanism by which CSE inhibits platelet aggregation. We showed that CSE inhibited arachidonic acid–induced TXA _2_ production and that the inhibitory effect of CSE on collagen-induced aggregation was suppressed significantly in TP ^–/–^ platelets compared with the inhibitory effect in WT platelets ( Fig. 2 ). These results indicate that the inhibitory effect of CSE on platelet aggregation is derived from reduced TXA _2_ production. In EP _2_^–/–^ , EP _4_^–/–^ , or IP ^–/–^ platelets, the inhibitory effect of CSE on U-46619-induced aggregation was not significantly different from that in respective control platelets ( Fig. 3A , B ). Furthermore, CSE did not increase the content of cAMP in platelets ( Fig. 3C ). These results indicate that receptors coupling to G _s_ expressed in platelets, including inhibitory prostanoid receptors, do not participate in the inhibitory effect of CSE on platelet aggregation.

COX-1 is an enzyme that is responsible for TXA _2_ production in platelets. CSE inhibited COX-1 activity in a concentration-dependent manner, and the pattern of inhibition was uncompetitive ( Fig. 4 ). In this study, we measured the peroxidase activity of COX, namely, the activity for conversion of PGG _2_ to PGH _2_ , the direct precursor of TXA _2_ . Our results indicated that CSE binds to the COX-1–PGG _2_ complex and stabilizes it, resulting in an uncompetitive inhibition of PGH _2_ production. In contrast to the inhibitory action on COX-1 activity, CSE enhanced TX synthase activity in a concentration-dependent manner ( Fig. 5 ). These results suggest that the inhibitory action of CSE on COX-1, an upstream enzyme of TX synthase in the arachidonic acid cascade, overcame the stimulatory action of CSE on TX synthase, leading to reduced TXA _2_ production in platelets. Like the action of CSE, it has been shown that catechol inhibited arachidonic acid–induced platelet aggregation and that the inhibition of COX activity and TXA _2_ production by catechol played a role in its antiplatelet effect. 20 Because catechol is included in the particulate phase of cigarette smoke, 16 21 it does not participate in the inhibitory effect of CSE, gas phase constituents of cigarette smoke. Further study is needed to determine what constituent(s) of CSE is responsible for the inhibitory effect of CSE on platelet aggregation.

Notably, CSE also inhibited the activity of COX-2 ( Fig. 4A ), a well-known player in inflammatory responses. On the other hand, it has been shown that CSE induces COX-2 expression in dendritic cells 38 and in tracheal smooth muscle cells. 39 Therefore, it is an interesting issue whether cigarette smoking increases or decreases COX-2-dependent prostanoid production under various inflammatory conditions in vivo.

This is the first report showing that CSE, the gas phase constituents of cigarette smoke, inhibits platelet aggregation and that its antiplatelet effect is derived from the inhibition of platelet COX-1 activity and resultant reduction in TXA _2_ production.
